# Mental Health Symptoms and Workplace Challenges among Australian Paramedics during the COVID-19 Pandemic

**DOI:** 10.3390/ijerph19021004

**Published:** 2022-01-17

**Authors:** Katherine Petrie, Natasha Smallwood, Amy Pascoe, Karen Willis

**Affiliations:** 1 Black Dog Institute, School of Psychiatry, University of New South Wales, Sydney 2000, Australia; Katherine.Petrie@unsw.edu.au; 2 Department of Allergy, Immunology and Respiratory Medicine, The Alfred Hospital, Monash University, Melbourne 3004, Australia; natasha.smallwood@monash.edu (N.S.); amy.pascoe@svha.org.au (A.P.); 3 Public Health, College of Health and Biomedicine, and Institute for Health and Sport, Victoria University, Melbourne 3011, Australia

**Keywords:** paramedics, pandemic, COVID-19, mental health, occupational stress, workplace, mixed methods

## Abstract

Background: Paramedics are vital to the health system response to the COVID-19 pandemic; however, the pressures on this workforce have been intense and challenging. This study reports on mental health symptoms and the working environment among Australian paramedics during the COVID-19 pandemic and explores their experiences of work and wellbeing during this time. Methods: An anonymous, online survey of frontline healthcare workers examined work environment, psychological wellbeing, and contained four open-ended qualitative items. Using a mixed method approach, quantitative data were analysed descriptively and qualitative data were analysed using content analysis. Results: This paper reports findings from 95 paramedics who provided complete quantitative data and 85 paramedics who provided free-text responses to at least one qualitative item. Objectively measured mental health symptoms were common among paramedics, and almost two thirds of paramedics self-reported experiencing burnout. Qualitative analysis highlighted key issues of safety and risk in the workplace, uncertainty and upheaval at work and at home, and lack of crisis preparedness. Qualitative analysis revealed four themes; ‘the pervasiveness of COVID-19 disruptions across all life domains’; ‘the challenges of widespread disruption at work’; ‘risk, uncertainty and feeling unsafe at work’, and ‘the challenges of pandemic (un)preparedness across the health system’. Conclusions: The COVID-19 pandemic resulted in considerable occupational disruption for paramedics and was associated with significant negative impacts on mental health. Findings emphasise the need for more adaptive working conditions, mental health support for paramedics, and enhanced crisis preparedness across the health system for future crises.

## 1. Introduction

Public health crises such as the COVID-19 pandemic represent a major threat to the wellbeing of frontline healthcare workers (HCW), including paramedics. Frontline HCWs working in the community are particularly affected by such events, which intensify existing stressors and create additional challenges, such as increased uncertainty in the workplace [1,2]. Paramedics are a vital and fundamental part of this frontline healthcare response to COVID-19 and have faced increased risk and workplace stress during the pandemic [3]. Paramedics have had to adapt quickly to multiple challenges, including fear of contracting the virus, increased workloads, insufficient resources and large amounts of new and constantly changing information. The work of paramedics is also impacted by changes to healthcare delivery in both primary and secondary care as well as community perceptions about accessing healthcare services generally. Despite an initial reduction in callouts during the pandemic, lockdown restrictions are implicated in increased callouts for psychiatric, behavioural and social reasons. Paramedics also bear the brunt of hospital protocol changes that may lengthen wait times for admission, and even the shift from face to face to telehealth in general practice may affect the type of call outs to which paramedics must respond [4]. Together, these conditions and changes pose significant threats to the wellbeing of paramedics, with potential for adverse effects on their mental health. 

Even outside of public health crises, evidence suggests that paramedics may be at increased risk of mental health problems. Whilst elevated rates of mental health disorders, particularly post-traumatic stress disorder (PTSD), have been identified among emergency service workers [5], a 2012 review suggested that ambulance personnel may be at particularly high risk for PTSD compared to other emergency services [6]. A systematic review and meta-analysis of common mental disorders estimated pooled prevalence rates of 27% for general psychological distress, 15% for depression, 15% for anxiety and 11% for PTSD amongst ambulance personnel worldwide [7]. 

There is evidence from previous crises, such as the severe acute respiratory syndrome (SARS) pandemic, that working during a pandemic can have long-term adverse mental health effects [8,9,10]. Meta-analyses of studies undertaken with HCWs early in the COVID-19 pandemic indicated that between 20 and 25% of HCWs reported high levels of anxiety, depression and post-traumatic stress symptoms [11,12]. International evidence has continued to emerge showing high rates of anxiety, depression, PTSD [13,14] and burnout [15,16] among HCWs working during the COVID-19 pandemic. Recent data from Australia have also demonstrated the substantial mental health burden of being a HCW during the pandemic [17,18]. However, paramedics are under-represented in research on the impacts of COVID-19, as well as in the literature on the mental health of HCWs more broadly [6,7], with Australian research particularly lacking. 

This study reports a subset of findings from the Australian Frontline Health Worker Study, which investigated the severity and prevalence of mental health problems and assessed the workplace, social and financial disruptions and challenges experienced by Australian HCWs during the COVID-19 pandemic [17]. A mixed methods sub-study was designed to focus on paramedics and had three objectives: (i) to assess the rates and severity of mental health problems and describe the occupational, social and financial disruptions experienced since the pandemic began; (ii) to identify the challenges and working conditions reported by paramedics during the pandemic; and (iii) to explore the experience and impact of these workplace factors on paramedics’ wellbeing and life. 

## 2. Materials and Methods

### 2.1. Study Setting, Design, Recruitment and Procedure

The full study methodology has been published elsewhere [17]. In summary, a nationwide, voluntary, cross-sectional, online, anonymous survey was conducted between 27 August and 23 October 2020. At this time, Australia was experiencing a second wave of the pandemic and in the state of Victoria, tough lockdown restrictions were imposed, restricting movement outside of the home, limiting the radius of travel, and requiring the use of masks in settings outside the home. While the situation has changed since then with lockdown restrictions eased and the Omicron variant of the virus spreading rapidly throughout most parts of Australia, in mid-2020, Victoria experienced far higher community transmission of the virus than other states, with active cases peaking at 6776 in early August [4], and these stringent lockdown restrictions were aimed at suppressing the spread of the virus.

Self-identified frontline HCWs from all health roles working in primary or secondary care across Australia were invited to participate using multiple recruitment and advertisement strategies. The study was promoted through health service organisations, and advertised widely via newspaper, television and radio news items, and social media sites. Strategies to recruit paramedics included contacting key ambulance personnel and asking them to promote the study to paramedics. 

Data were collected at a single time point and each participant completed the survey once. Online consent was obtained prior to commencing the survey. The survey comprised: demographics, work environment, self-reported mental health issues and five objective validated mental health measures (the Generalised Anxiety Disorder (GAD-7) for anxiety [18], Patient Health Questionnaire (PHQ-9) for depression [19], abbreviated Impact of Event Scale (IES-6) for post-traumatic stress disorder (PTSD) [20], abbreviated Maslach Burnout Inventory (MBI) for burnout, and abbreviated 2-item CD-RISC-2 scale for resilience [21]). Four optional open-ended free-text qualitative items were included asking participants about (a) what would help them to deal with stress and anxiety; (b) the main challenges of the pandemic; (c) the strategies they thought that would be useful in the future; and (d) if there was anything else they wished to add. Participants could write as much or as little as they wished in their responses. 

### 2.2. Ethics Approval

The study received ethics approval from the Melbourne Health Human Research Ethics Committee (HREC/67074/MH-2020 approved 20 August 2020). All participants provided consent online. Information for participants was provided on the survey website, indicating that data would be stored securely, that no identifiable data were being collected, and that care would be taken to ensure that data remained anonymous in reporting. As the survey was about the psychosocial impact of COVID-19, links to mental health resources were also provided on the survey website.

### 2.3. Sub-Study Participants and Data Analysis

As paramedics were the focus of this sub-study, the current study reports only on participants who self-identified as paramedics. Quantitative data analysis was performed by two authors (KP and AP) and checked by NS, using SPSS statistical software version 26.0 (IBM Corp., Armonk, NY, USA). Demographic and work characteristics, mental health symptoms and workplace changes during the pandemic are reported descriptively, with Chi-square tests of independence (categorical data) and independent *t*-tests (continuous data) performed to compare paramedics with all other HCWs. Statistical significance was set at *p* < 0.05. 

A qualitative descriptive approach [22,23] using content analysis was used to analyse the free-text responses. Data were exported into Excel, and an inductive approach was taken to elicit the key ideas in the responses. All responses were coded initially by one author (KP), with up to three codes applied per response, and a codebook was developed, with coding and codebook reviewed weekly by KW. As coding proceeded, additions were made to the codebook with the emergence of new ideas, and some coding categories were condensed or removed as coding progressed. Codes with similar or related underlying ideas were grouped together into key themes [24,25]. Weekly review meetings between KW and KP focused on achieving consensus on themes and sub-themes, with NS providing critical feedback on coherence between codes and themes at two time points.

## 3. Results

From 9518 participants, complete data were received from 7845 (82.4%), of whom 95 (1.2%) were paramedics. Half of all paramedics were female (50.5%, *n* = 48) and most were aged between 31 and 50 years (57.9%, *n* = 55) (Table 1). While the study was promoted nationally, most participants were from the Australian state of Victoria, partially explained by the fact that this was where there was the highest number of community transmissions and the most severe lockdown restrictions during the period of the survey. 

Most paramedics were working full-time, and the majority reported working with individuals infected with COVID-19 (81.1%, *n* = 77). Concern about possible transmission of COVID-19 to their family was high (82.8%, *n* = 77) (Table 2). Almost half of paramedics reported working increased hours (49.4%, *n* = 47). Almost half of participants indicated a need for more training regarding personal protective equipment (PPE) or managing people infected with COVID-19 (45.3%, *n* = 43).

One third of participants reported having a diagnosed mental health condition before the pandemic (33.7%, *n* = 32) (Table 3). Many participants self-reported experiencing subjectively determined mental health issues since the onset of the pandemic, most commonly symptoms of burnout (61.1%, *n* = 58) and anxiety (53.7%, *n* = 51). Paramedics’ responses to objective mental health symptom scales indicated moderate to severe symptoms of: emotional exhaustion (burnout subscale) (72.6%; *n* = 69), post-traumatic stress (42.1%; *n* = 70), anxiety (30.5%; *n* = 29) and depression (26.3%; *n* = 25). Paramedics reported significantly higher rates of self-reported PTSD (14.7%) compared to other HCWs (5.3%) (*p* < 0.001) and significantly higher rates of moderate to severe depersonalisation symptoms on the burnout scale compared to other HCWs (54.7% vs. 37.2%, respectively) (*p* < 0.001). 

### 3.1. Qualitative Findings 

Of 95 participants, 84 paramedics provided valid free-text responses to at least one of the four qualitative items (88.4% of sample in quantitative analysis). Gender and work location (see Table 4 below) generally approximated the distribution in the sample overall; and the breakdown of responses to individual questions was the same as the sample overall, with Question 2 being the most frequently answered and Question 4 being the least frequently answered. Over half of participants provided responses to three or four of the free-text questions (*n* = 51); 26 participants responded to two questions; and 7 participants answered one question only.

Responses varied considerably, with some writing a single word, but most participants wrote a paragraph or more about their experience, and many covering multiple points within a single answer. Less than 10 responses per question were 1–2 words only; but these still provided rich data. Brief responses such as ‘wearing PPE’, ‘burnout’, and ‘management caring’ indicating challenges of the pandemic; and ‘rest breaks’, ‘more compassion’, ‘more resources’ and ‘better plan’ indicating the strategies useful to assist healthcare workers in future crises, were all covered by other participants in longer, more detailed responses. The data also varied according to whether participants wrote about the same issue (e.g., the need for more time off) across multiple questions or noted quite different issues in their responses to different questions. 

From these rich and diverse approaches to the free-text questions, four themes related to paramedics’ experiences of working and living during the early COVID-19 pandemic were identified. These were: the pervasiveness of COVID across all life domains; the challenges of widespread disruption at work; risk, uncertainty and feeling unsafe at work; and the challenges of pandemic (un)preparedness across the health system. Illustrative quotes are presented for each theme (Table 5, Table 6, Table 7 and Table 8) to exemplify the key ideas in the data. 

### 3.2. Theme 1: The Pervasiveness of COVID across All Life Domains

Paramedics described how the COVID pandemic had generated widespread disruption and a sense of fear that permeated all domains of their life, including work, home, personal and social contexts, particularly when usual supports and resources, such as childcare, were unavailable (Table 4). This was evident in four ways. First, they described the struggles to meet increased demands of both work and their personal life (such as caring responsibilities), meaning they were no longer able to achieve a work/life balance. Second, they described the pervasiveness of pandemic stress in their everyday life, being impossible to escape the pandemic at work or at home, resulting in an inability to ‘switch off’ from the pandemic. Third, the physical and mental effects of the pandemic were described and paramedics reported feelings of increased stress and burnout, commonly using words such as “exhaustion”, “burnout” and “fatigue” to describe their experience. Finally, paramedics struggled to draw on their usual coping strategies, including social support, in order to self-care. Many reported that being unable to engage in their usual self-care strategies was the main challenge to their mental health and prevented them from effectively managing the emotional and physical challenges of working and living through a pandemic. The loss of social support from family and friends as a result of lockdown restrictions was frequently mentioned as a major loss that negatively affected their wellbeing. In particular, for those living alone, the challenges of isolation and lack of social connection were particularly pronounced and associated with considerable concern and fear around how they would cope by themselves should they be forced to quarantine. As one paramedic reflected, all of this meant that “normal will never be normal again” (male, age 41–50). 

### 3.3. Theme 2: The Challenges of Widespread Disruption at Work 

Paramedics wrote extensively about the impact of highly disrupted working conditions and described a number of major challenges in an unpredictable workplace with working conditions that were more physically and emotionally demanding (Table 6). They described increased workloads, more complex duties, longer working hours in uncomfortable, hot PPE, and undertaking additional time-consuming tasks such as ambulance cleaning that often resulted in unwanted overtime. 

Constantly changing procedures and information was described as a major source of stress as paramedics struggled to keep up with rapid changes in their day-to-day role and guidelines, which occurred “daily”, “hourly” and/or “mid-shift”. They described working in an environment where they frequently encountered inconsistencies, including in the information employees were receiving from their workplace, and in the approach and protocols followed by different health services.

Throughout their responses, paramedics described issues with communication. Examples included the use of multiple channels of communication, none of which were universally used by all employees, nor by management, and none of which held all the necessary information paramedics required. Some described overload of information, with lots of unnecessary and non-essential information. Others described a lack of communication, with insufficient or incomplete information that was often contradictory. Paramedics wrote about a sense of confusion at work as a result of this inconsistency in communication, some describing a fear of being left behind, and others describing how each employee had different interpretations of the guidelines. They called for streamlined centralised communication of selective, clear and consistent information from the organisation, with regular (but not too regular) updates. This needed to be accessible to all employees with clear outlines of rules, procedures and standards. 

Paramedics described wanting a more proactive, responsive and accountable leadership approach from management. Some desired greater dialogue with management, where leaders were available and accessible to staff, and listened to and addressed paramedics’ needs and suggestions. Paramedics wrote about feeling unsupported at work. Some felt that they were not listened to or respected by management, which contributed to a perceived “reality gap” between management and the frontline worker. Some reported a lack of direct contact from management to check in or to listen to employees, whilst others described feeling unsupported in terms of not being adequately recognised for the increased and intensified workload they had taken on or for placing themselves in high-risk situations daily. Many paramedics described feeling that their mental health needs were not supported by the organisation. They recommended that workplaces should provide proactive and timely support to address mental health needs. Suggested strategies included appointments with mental health professionals through work, online counselling, increased number of Medicare-funded sessions with psychologists, peer support and manager support programs. They wanted management to check in on staff wellbeing, and work conditions that included additional paid pandemic sick leave and gestures of appreciation, such as provision of food and drinks at work. Paramedics discussed the value of informal mental health supports at work, mostly through debriefing with colleagues and team members.

### 3.4. Theme 3: Risk, Uncertainty and Feeling Unsafe at Work 

The third theme focused on risk, uncertainty and feeling unsafe at work (Table 7). Feelings of being unsafe and unprotected at work were raised most frequently in relation to access to appropriate, quality PPE, including masks, Tyvek suits, and hair nets. Feeling unsafe at work was also heightened by having to work in spaces or facilities where social distancing was impossible, such as in enclosed ambulances, often for long periods of time. Lack of PPE exacerbated fear and worry regarding the risk of infection, both to themselves and their loved ones. Inadequate PPE left paramedics feeling physically exposed and unprotected. They felt that their safety was not prioritised, and this was exemplified by inadequate or lack of PPE. Some paramedics felt that inflexible procedures and work demands left them feeling unnecessarily exposed to risk. Some described feeling unsafe due to being unable to make day-to-day decisions that ensured their right as an employee to be safe at work and that were appropriate to the context of working in a highly infectious viral pandemic. These issues were all connected to the broader difficulty of balancing risk of exposure and their own safety with their professional duty to provide quality patient care, a challenge most often raised in relation to high-risk clinical situations. Many paramedics described a sense of uncertainty and unpredictability at work, particularly in relation to the ‘unknown’ risk and unprecedented situations they faced in the pandemic.

### 3.5. Theme 4: The Challenges of (Un)Preparedness across the Health System

For many paramedics, the issues they identified in the workplace were emblematic of, and often compounded by, similar problems in the health system (Table 8). Paramedics described a lack of pre or forward planning for a crisis at work and in other services, and widespread unpreparedness for a public health crisis across the health system. They described how an already understaffed health system now needed to accommodate extra duties such as cleaning and COVID-19 testing, or to off-set staff losses due to quarantine. This lack of preparedness and resources in their immediate workplace, and in the hospitals they were sent to, and exacerbated the stress and pressure for paramedics as they struggled to manage increased workloads with inadequate resources. They described how working in an unprepared public health system exacerbated their sense of feeling unsafe and unprotected at work. 

Paramedics also wrote about the reactive and slow responses to new knowledge, rather than a proactive response, often in relation to how knowledge about the transmission of the virus (e.g., airborne) improved, but was not reflected in their PPE. For example, they were concerned that only base levels of PPE were first recommended, and these were then only upgraded as evidence changed, rather than implementing universal high-tier protection and the precautionary principle to exposure from the pandemic outset first. Some described a lack of faith in the government or in ambulance management to keep them safe and to keep abreast of the latest scientific evidence. 

## 4. Discussion

The experiences of working and living in the COVID-19 pandemic exacerbated work-based challenges and posed additional adverse impacts on paramedics’ mental health. The majority of paramedics reported mental health issues, most commonly symptoms of burnout and anxiety. Whilst there is limited evidence assessing mental health outcomes in paramedic-only samples during public health crises, our findings are consistent with evidence from reviews [11,26], and overseas and Australian studies [12,17,27,28,29,30] showing the negative mental health impacts of working as a HCW during the COVID-19 and SARS pandemics [31,32]. 

Despite this psychological burden, paramedics described a general lack of support from their workplaces for their mental health needs, and a culture of disregarding their needs. In the absence of support from management, paramedics described turning to their team members for support, as observed in other HCW groups such as nurses [33]. The importance of workplace support as a protective factor for mental health and a buffer for workplace stress is now well-recognised [24,34], and emerging evidence has identified this pattern in ambulance personnel [35]. Evidence from past [36] and current pandemics [2,37,38] suggests organisational and collegial support may serve a protective role for HCW mental health. Efforts to enhance support at work for paramedics warrant further investigation. 

A salient finding was that paramedics reported significantly higher rates of self-reported PTSD, and of moderate to severe depersonalisation symptoms on the burnout scale, compared to other HCWs. Figures are in excess of estimated PTSD rates among ambulance personnel reported in a meta-analysis of studies conducted in non-crisis times [7], suggesting that the pandemic was associated with this symptom burden. A review of work-related factors for PTSS and PTSD among HCWs during past pandemics and COVID-19 [39] identified key risk factors that were also recounted by paramedics in our study, including high exposure to the virus, high perceived threat to health or life, and social isolation. Protective factors included supervisor and colleague support, perceived adequacy of training and positive work aspects including communication—factors that were described as lacking by many paramedics. Additionally, systemic workplace issues such as increased workloads, frequently changing protocols and inadequate PPE have been directly associated with burnout and distress in HCWs [40,41]. Together, this indicates that addressing organisational and systemic factors during a pandemic may be a promising strategy to improve mental health outcomes in paramedics and other HCWs [42]. 

Paramedics described major occupational uncertainty and upheaval during the pandemic, feeling unsafe at work, felt unsupported by management, and working within organisations and a health system that was unprepared for a crisis. Disruption, uncertainty and stress negatively impacted on emotional, social and physical wellbeing for most paramedics. They recounted widespread occupational disruptions including unpredictability in the workplace, inconsistent information, and rapid changes to the workplace, and cited adverse working conditions—occupational challenges also reported in qualitative studies with other HCWs during COVID-19 and past pandemics [28,43,44,45]. 

The most common workplace challenges for paramedics were problems with communication, and inadequate support from management. For some, this working environment exacerbated feelings of being unsafe and unprotected at work and created difficulties in balancing patient and personal safety in high-risk clinical situations. A lack of pandemic preparedness at work and in the health system was a major challenge, and paramedics recommended a proactive, evidence-based, coordinated and consistent approach be implemented across the health system in future crises. 

Based on our findings, other studies [28] and commentary [46], strategies to enhance pandemic preparedness and supports for paramedics in future crises should include improvements to working conditions, training, PPE supply, consistent communication, more effective leadership and additional mental health support at work. Better health system preparedness through consistent implementation of guidelines, and additional training and resourcing of equipment and staff are needed to manage increased demand during a crisis. Further research with paramedics is warranted to understand the long-term impact of working in a pandemic on their wellbeing and working conditions and to develop targeted strategies to enhance occupational safety and support.

The small sample size, cross-sectional survey design and potential for self-selection and response bias are limitations of this study. Nevertheless, the sample size achieved is similar to other similar studies [47,48]. The findings may not be representative of the experiences and views of all Australian paramedics; however, there is congruence between our findings and that of other Australians studies drawn from different geographic areas [28]. Anonymous online surveys may have a sample selection bias, as they provide an avenue for participants to disclose mental health distress, particularly when distress is stigmatised. Conversely, the strength of this approach is that it provides a safe environment for the disclosure of such feelings. The strengths of the study include its mixed methods approach to understand the experience of paramedics, comprising the use of standardised validated mental health questionnaires and specific COVID-19-related items, along with free-text questions where paramedics could describe their experiences.

## 5. Conclusions

This study describes the experiences and challenges faced by paramedics working in the COVID-19 pandemic in Australia, particularly within the state of Victoria. The prevailing message conveyed by paramedics was the need for a safe, consistent, prepared, adaptive workplace where they felt supported to stay mentally healthy and to provide high-quality patient care during the pandemic.

## Figures and Tables

**Table 1:** Participants’ characteristics (*n* = 7845).

Characteristic	Paramedics (*n* = 95)		All Other Professions (*n* = 7750)	
	Frequency (*n*)	%	Frequency (*n*)	%
Age (years)				
20–30	22	23.2	1838	23.7
31–40	32	33.7	2218	28.6
41–50	23	24.2	1715	22.1
>50	18	18.9	1979	25.5
Gender				
Male	45	47.4	1413	18.2
Female	48	50.5	6295	81.2
Non-binary	1	1.1	18	0.2
Prefer not to say	1	1.1	24	0.3
Living arrangements				
Lives alone	15	15.8	1072	13.8
Children <16 years at home	37	38.9	2707	34.9
Persons aged >65 years at home	9	9.5	688	8.9

**Table 2:** Workplace environment (*n* = 7845).

Characteristic	Paramedics (*n* = 95)		All Other Professions (*n* = 7750)		
	Frequency (*n*)	%	Frequency (*n*)	%	*p*
Location of practice					<0.001
Metropolitan	47	49.5	6325	81.6	
Regional/remote	48	50.5	1425	18.4	
Current employment status					<0.001
Full time	81	85.3	3736	48.2	
Part time	9	9.5	3633	46.9	
Casual/other	5	5.3	381	4.9	
Any change in working hours since the pandemic commenced **					
Increased paid hours	31	32.6	1603	20.7	0.004
Increased unpaid hours	16	16.8	1670	21.5	0.27
Decreased paid or unpaid hours	4	4.2	862	11.4	0.03
No change	52	54.7	3986	51.4	0.52
Currently working with people infected with COVID-19	77	81.1	2986	38.5	<0.001
Received training on PPE during the pandemic	88	92.6	5049	65.1	<0.001
Confidence in using PPE (mean score) *	5.30 (*n* = 93)	SD = 1.79	5.38 (*n* = 5860)	SD =1.60	0.65
Received training to care for patients with COVID-19	48	50.5	2744	35.4	<0.01
Confidence in caring for people with COVID-19 (mean score) *	5.12 (*n* = 91)	SD = 1.63	4.89 (*n* = 5786)	SD = 1.58	0.16
Desires more training regarding PPE or managing people with COVID-19	43	45.3	2957	50.5	0.42
Worried their role will lead to them transmitting COVID-19 to family					0.18
Not worried	11	11.8	718	12.3	
Neutral	5	5.4	669	11.4	
Worried or very worried	77	82.8	4473	76.3	
Worried about being blamed by colleagues if they contract COVID-19					0.28
Strongly or somewhat agree	62	65.3	4886	63.0	
Neither agree/disagree	19	20.0	1256	16.2	
Strongly or somewhat disagree	14	14.7	1608	20.7	
Communication received from the workplace during the pandemic has been useful and timely					0.79
Strongly or somewhat agree	69	72.6	5763	74.4	
Neither agree/disagree	9	9.5	792	10.2	
Strongly or somewhat disagree	17	17.9	1195	15.4	
Believed their workplace actively supported their wellbeing and mental health during the pandemic					0.77
Strongly or somewhat agree	63	66.3	5288	68.2	
Neither agree/disagree	14	14.7	1205	15.5	
Strongly or somewhat disagree	18	18.9	1257	16.2	

* Confidence was scored on a 7-point Likert scale: 1 = very unconfident, 7 = very confident. ** Multiple responses could be selected.

**Table 3:** Mental health symptoms (*n* = 7845).

Characteristic	Paramedics (*n* = 95)		All Other Professions (*n* = 7750)		
	Frequency (*n*)	%	Frequency (*n*)	%	*p*
Pre-existing mental health condition	32	33.7	2357	30.4	0.49
Self-reported mental health issues experienced since COVID *					
Anxiety	51	53.7	4824	62.2	0.09
Burnout	58	61.1	4517	58.3	0.59
Depression	33	34.7	2142	27.6	0.13
PTSD	14	14.7	413	5.3	<0.001
Other mental health problems	8	8.4	320	4.1	0.04
No mental health issues	17	17.9	1413	18.2	0.93
Mental health issues assessed by validated scales					
Burnout—Depersonalisation (DP)					<0.001
Low	43	45.3	4767	62.8	
Moderate/High	52	54.7	2825	37.2	
Burnout—Emotional Exhaustion (EE)					0.71
Low	26	27.4	2216	29.1	
Moderate/High	69	72.6	5389	70.9	
Burnout—Personal Accomplishment (PA)					0.39
Low	33	34.7	2325	30.6	
Moderate/High	62	65.3	5268	69.4	
Anxiety—GAD7					0.62
None to Mild	66	69.5	5559	71.8	
Moderate to Severe	29	30.5	2188	28.2	
Depression—PHQ9					0.97
None to Mild	73	76.8	5919	76.7	
Moderate to Severe	22	23.2	1801	23.3	
Impact of events/trauma—IES6					0.75
None to Mild	55	57.9	4585	59.5	
Moderate to Severe	40	42.1	3115	40.5	
Resilience—CD-RISC-2 (mean score)	3.18 (*n* = 94)	SD = 0.64	3.22 (*n* = 7746)	SD = 0.66	*t*-test 0.65

* Multiple options could be chosen. Anxiety (GAD-7): 0–4 = none/minimal, 5–9 = mild, 10–14 = moderate, 15–21 = severe anxiety; Depression (PHQ-9): 0–4 = none/minimal, 5–9 = mild, 10–14 = moderate, 15–19 = moderately severe, 20–27 = severe depression (Kroenke et al., 2001); PTSD (IES-6): 0–9: none-mild, >9 moderate-severe (Thoresen et al., 2009); Burnout on the MBI is indicated by higher scores on the EE and DP, and lower scores on the scale of PA. Burnout subdomains (MBI): depersonalisation DP: 0–3 = low, 4–6 = moderate, 7–18 = high; emotional exhaustion EE: 0–6 = low, 7–10 = moderate, 11–18 = high; personal accomplishment PA: 0–13 = low, 13–14 = moderate, 15–18 = high (Rikey et al., 2018).

**Table 4:** Responses to free-text questions.

Question	Gender *		Work Location	
	Female	Male	Metropolitan	Regional
1. What do you think would help you most in dealing with stress, anxieties, and other mental health issues (including burnout) related to the COVID-19 pandemic? (*n* = 61)	37	24	29	32
2. What did you find to be the main challenges that you faced during the COVID-19 pandemic? (*n* = 81)	46	34	42	39
3. What strategies might be helpful to assist frontline healthcare workers during future crisis events like pandemics, disasters, etc.? (*n* = 69)	37	31	38	31
4. Is there anything else you would like to tell us about the impact of the COVID-19 pandemic or regarding supports that you feel are useful for well-being? (*n* = 26)	14	12	8	18

* Questions 2 and 3 also include response from one participant who indicated ‘prefer not to say’.

**Table 5:** Theme 1: The pervasiveness of COVID across all life domains.

Work–life imbalance challenges	Not being able to spend time with family as I was working. (male, age 31–40) Balancing work/home life. (female, age 31–40) Unrealistic expectation to be available to my staff 24/7 whilst having a young family. (female, age 31–40)
Pervasiveness of COVID stress in everyday life	[I could never] “get away” from the pandemic stressors. Affected at work then again at home due to isolation and restrictions. (male, age 31–40).
Physical and emotional symptoms	[Main challenge was] fatigue, physical effects of PPE e.g., dehydration, headaches. (female, age 50–64) [Main challenge was] exhaustion from cleaning, difficulties with eye allergies from chemicals used, heat exhaustion from full PPE. (female, age 41–50) [I have] worsening mental health and increased burn out. (female, age 31–40)
Changes to coping strategies and lack of social support	Social isolation, not being able to travel, as travel was my time away and a de-stressor. Unable to connect with other people other than who I work with. (female, age 31–40) [Feeling] over worked, burn out, no avenues for stress relief. (female, age 20–30)
Strategies needed to support mental health	Access to health facilities, i.e., pools/gyms, etc. (male, age 41–50) Allow persons of single households to connect with another household much earlier on. Being able to access psychology appointments straight away (female, age 31–40).

**Table 6:** Theme 2: The challenges of widespread disruption at work.

Disrupted, difficult working conditions	Added complexity and the volume of work and change in a short time is exhausting to keep up with. (female, age 50–64) [Main challenge was] Cleaning ambulances and equipment in trucks post COVID cases! (female, age 31–40)
Working with inconsistencies	Biggest issues are inconsistent approaches between hospitals and having to follow different protocols depending on where patients are transported. Hard to know what we are doing at times as it changes so often, generally mid shift with no notification. (female, age 41–50) [Main challenge was] Keeping up with organisational changes in relation to PPE, workplace procedures and changes to practice. (female, age 20–30)
Communication work challenges	[My service] have relied heavily upon “Workplace” which is a Facebook style social media platform that not all staff access. (male, age 20–30) Information has been given over multiple platforms, e.g., [we] must be using Workplace and checking emails and intranet as not all information was provided on both. Information being passed on mid shift about changes was inadequate, we were reliant on colleagues to see the information. There was also use of terms early on which we had never heard and weren’t explained, e.g., told to consider social distancing mid shift but no direction of what that meant. (female, age 31–40) Getting consistent information from management has been tough. (female, age 20–30)
Supportive management	[We need] Less management demands at work regarding KPIs [key performance indicators] and [better] PPE management and constant updates about changes and policy alterations. it’s a stressful time and I feel management are adding to our stress more when not needed. (male, age 41–50) [We need] More information and support from management. (female, age 31–40)
Abandoned and disillusioned	I feel totally abandoned by my employer and I’m on my own to manage my own mental health. I have seen so many front line worker collapse emotionally and be treated that bad they leave or kill themselves. Lift your game. (male, age 41–50) [Main challenge was] lack of support. Lack of respect. Lack of listening skills for staff to get their grievance out. Staff identify [name of service] as best care for the lowest price. They concentrate on compliance to appropriate documentation and KPI. Forget about mental health, emotional support, wellbeing, physical health and family with female staff on FWA [Flexible Working Arrangements], parental concerns. (male, age 50–64)
Workplace support for mental health	[We need] manager support, proactive support from psychologist or peer support services. (female, age 20–30) Provide us with sufficient leave that doesn’t come out of sick leave when we try to do the right thing. More one on one support (female, age 41–50). Shorter shifts, calling and checking in on staff. (female, age 20–30).

**Table 7:** Theme 3: Risk, uncertainty and feeling unsafe at work.

Feeling unsafe at work	[I have] constant fear of becoming infected and passing on to family (female, age 20–30) [Main challenge was] face to face contact with an unknown risk on a day to day basis. (male, age 41–50)
The need for PPE	Anxiety in the workforce was in direct response to concern over appropriate/changing and supply of PPE. (female, age 50–64)
The challenges of balancing safety and patient care in high-risk workplace situations	Upper management [need to] take on suggestions that still provide best evidence based care for the patient and keep them safe BUT do not necessarily expose health care workers to risk. (female, age 41–50) During a pandemic health care workers need to be able to make decisions that are outside of the norm. For example—refusing to transport a person in confined space showing signs and symptoms of Coronavirus that are otherwise well enough to manage themselves at home with phone consultation and monitored health care checks, etc. (female, age 41–50)
Unpredictability of day-to-day work	[Main challenge was] Not knowing what we would be walking into on a job (female, age 20–30)

**Table 8:** Theme 4: The challenges of pandemic (un)preparedness across the health system.

Lack of resourcing and training at work	Consider extra training and resources prior to peak staff isolating and employ additional staff well before the requirement. (female, age 20–30)
Inadequate resourcing and training in the health system	[We need] Earlier identification and risk management planning. Better to go hard on risk management early such as PPE and training than have to play catch up later when people are already facing exposures. (male, age 31–40)
Reactive versus proactive approach across the organisation and the health system	[We need] the PPE to be highest level first then reduce if evidence supports. We went from lower to higher levels of PPE as evidence changed. Also case definition for suspected COVID-19 went from conservative to almost anyone. These two issues in combination could have been disastrous and never made sense so this was most stressful stage to work. (male, age 31–40) Better forward planning from healthcare organisations. A lot of making it up as they went along was happening as they didn’t have systems in place for quarantine, cleaning, what to do in case of a breach (female, age 20–30) Knowing the government has a strategic plan in place to best deal with future incidents. I felt the government were ill prepared and were required to chase their tail the whole time. I lost faith in the government’s ability. (male, age 31–40)

## Data Availability

Data available upon reasonable request to the corresponding author.
